# The Utilization of *Bacillus subtilis* to Design Environmentally Friendly Living Paints with Anti-Mold Properties

**DOI:** 10.3390/microorganisms12061226

**Published:** 2024-06-18

**Authors:** Yuval Dorfan, Avichay Nahami, Yael Morris, Benny Shohat, Ilana Kolodkin-Gal

**Affiliations:** 1 Faculty of Electrical Engineering, Holon Institute of Technology, Holon 5810201, Israel; avichaynahami@gmail.com (A.N.); shohatbenny@gmail.com (B.S.); 2 The Scojen Institute for Synthetic Biology, Reichman University, Herzliya 4610101, Israel

**Keywords:** anti-mold, paint, *Bacillus subtilis*, probiotics, anti-fungal, *Aspergillus niger*, non-ribosomal peptides, bio-convergence

## Abstract

The anti-fungal properties of the probiotic bacterium *Bacillus subtilis* have been studied extensively in agriculture and ecology, but their applications in the built environment remain to be determined. Our work aims to utilize this biological component to introduce new diverse anti-mold properties into paint. “Mold” refers to the ubiquitous fungal species that generate visible multicellular filaments commonly found in household dust. The development of mold leads to severe health problems for occupants, including allergic response, hypersensitivity pneumonitis, and asthma, which have significant economic and clinical outcomes. We here demonstrate the robust effect of a commercial paint enhanced with Bacillus subtilis cells against the common mold agent, *Aspergillus niger*, and identify three biosynthetic clusters essential for this effect. Our results lay the foundation for bio-convergence and synthetic biology approaches to introduce renewable and environmentally friendly bio-anti-fungal agents into the built environment.

## 1. Introduction

In competitive environments, such as the soil, bacteria benefit other organisms, for example, *biocontrol agents* form biofilms on the surface of plant roots, thereby preventing the growth of bacterial and fungal pathogens [1,2]. Soil and plant-associated microbial populations survive in this highly competitive niche by producing a broad arsenal of antibiotics and evolving complex antibiotic-resistance mechanisms [3,4].

Antibiotic production was shown to be regulated and affected by biological, chemical, and physical inputs from the environment [5,6,7]. Members of the *Bacillus subtilis* clade contain several species of Gram-positive, soil-dwelling, beneficial bacteria that employ multiple strategies to compete with community members that share the same growth niche [8,9,10,11,12,13,14,15]. Two of the primary classes of antibiotics and antifungals produced by the *Bacillus* genus are the Non-Ribosomal Peptides (NRPs) and Polyketides (PKS), which are synthesized by large multi-enzyme complexes of non-ribosomal peptide synthetases (NRPSs) and polyketide synthases (PKSs) [8,16,17].

The plant growth-promoting bacterium and biocontrol agent *B. subtilis* [8,18,19] contains three different NRP biosynthetic clusters. These clusters are responsible for the biosynthesis of surfactin, bacilysin, and plipastatin. In addition, its genome carries an NRP/PKS hybrid biosynthetic cluster responsible for the biosynthesis of bacillaene [8,20,21,22,23]. These antibacterial substances contributed to the biocontrol properties of *B. subtilis* and were often reported for their antifungal properties, although their interaction with each other was only evaluated for other *Bacillus* species [24].

Surfactin is a small cyclic lipopeptide induced during the development of genetic competence [25,26] and was suggested to promote horizontal gene transfer [27]. The machinery for surfactin synthesis is encoded by the genes within the *srfAA–AB–AC–AD* operon [28]. Surfactin is a powerful surfactant with antibacterial [29,30] and antifungal properties [31]. Bacillaene and dihydrobacillaene [20,32] are linear antimicrobial macrolides with two amide bonds. They are synthesized by the PksC-R clusters mega-complex, composed of thirteen PKS and three NRPS modules [32]. Bacilysin is a non-ribosomal dipeptide composed of the amino acids L-alanine and L-anticapsin. This dipeptide acts as an antibiotic capable of inhibiting/eliminating multiple bacterial and fungal pathogens. The synthesis of this broad-spectrum peptide is controlled by the *bac* operon (*bacABCDE*) [33,34,35]) Lastly, plipastatin is synthesized non-ribosomally by five fengycin synthetases (*ppsA-E*) [36].

Plipastatin is an established anti-fungal agent with broad anti-fungal activities against *Fusarium graminearum* [36] and *Plasmopara viticola* [37]. However, the products of *bac*, *pps*, and *srf* biosynthetic clusters carry anti-envelope activities and, therefore, can potentially eliminate filamentous fungi efficiently [38,39]. This overlapping mode of action obtained by different chemistries suggests potential additive/synergistic anti-fungal interactions between the three clusters. In contrast, the polyketide antibiotic bacillaene was suggested to inhibit translation in an unknown mechanism, potentially indicating more specificity toward bacteria [40].

While the anti-fungal properties of *B. subtilis* were studied extensively in agriculture [8], their construction applications, and particularly anti-mold surfaces, are poorly studied. The term “mold” refers to the ubiquitous fungal species producing visible multicellular filaments. Mold formation can cause various human pathologies, including but not limited to allergic bronchopulmonary aspergillosis, allergic fungal rhinosinusitis, hypersensitivity pneumonitis, and asthma [41]. While living paints with *B. subtilis* for mold prevention were not evaluated previously, anti-corrosive paints based on *B. subtilis* and related specie were previously reported [42]. Therefore, developing environmentally friendly anti-mold surfaces and paints with *B. subtilis* bears economic and clinical importance.

As *Bacillus subtilis* is a probiotic agent, recognized as GRAS (generally recognized as safe) [43] for human consumption, we here asked whether we can harvest its antifungal properties to enhance the anti-mold properties of paints. Among our goals, we aimed to test whether the different antifungal agents encoded by this bacterium productively interact to increase their potency towards fungi. By using natural producers to develop sustainable biopesticides for the construction industry, we can reduce our reliance on current chemical solutions and greatly benefit human health.

## 2. Materials and Methods

**Strains and Media.** All experiments were performed with *B. subtilis* NCIB 3610 WT strain [44] and its mutant derivatives for antibiotic biosynthetic clusters described by us previously [24]. Growth experiments were performed in indicated media: LB broth (Difco, Le Pont de Claix, France), B4-Ca, and LBGM liquid medium (LB plus 1% [*v*/*v*] glycerol and 0.1 mM MnSO_4_) were prepared by us as described previously [24,45,46]. The composition of MSgg medium was 5 mmol/L potassium phosphate, 100 mmol/L MOPS pH 7, 2 mmol/L MgCl_2_, 50 μmol/L MnCl_2_, 125 μmol/L FeCl_3_, 700 μmol/L CaCl2, 1 μmol/L ZnCl_2_, 2 μmol/L thiamine, 0.5% glycerol, 0.5% glutamate, 50 μg/mL threonine, tryptophan and phenylalanine, as described previously [44,47].

The *Aspergillus niger* (*A. niger*) strain was kindly provided by Prof. Oded Yarden (The Hebrew University of Jerusalem, Rehovot Campus).

**Collection of the conditioned medium.** *B. subtilis* WT or indicated mutants were isolated on a solid LB plate, inoculated into 3 mL of LB broth, and grown overnight 30 °C on indicated media. The conditioned medium was centrifuged at 8000 rpm for 10 min; supernatant was removed and filtered through a 0.22 µm filter. The conditioned medium’s acidity was measured using the pH meter (Mettler Toledo) [48].

The growth-inhibiting 100% methanol elute of the overnight conditioned medium of *B. subtilis* was eluted from a C-18 SPE column (Waters, Milford, MA, USA) and evaporated to 10% of its initial volume using SpeedVac Vacuum Concentrator (Thermo Fischer Scientific, Waltham, MA, USA). Prior to the experiment, the eluate was diluted with Phosphate-buffered Saline (PBS) (Sigma Eldrich, St. Lewis, MI, USA).

***A. niger* spore preparation.** *A. niger* cells were grown for seven days at 28 °C via subculturing onto Sabouraud glucose agar (SGA) to obtain adequate sporulation. Conidia were collected with a cotton swab and suspended in saline with 0.05% Tween 20, as carried out previously in [49]. Heavy particles were allowed to settle, and the turbidity of the remaining culture was measured spectrophotometrically (OD = 530 nm) in a microplate spectrophotometer (Infinite^®^ 200 PRO Microplate Reader, Tecan LTD, Männedorf, Switzerland). The transmission was adjusted to correspond to 0.5–3 × 10^6^ CFU/mL. The viability was confirmed via plating serial dilutions onto LB plates. For inoculum preparation, the conidial suspensions were diluted 1:100 to obtain a final inoculum of 0.5–2 × 10^5^ CFU/mL.

**Evaluating the anti-fungal properties of commercial paints.** To inoculate the *A. niger* and generate a homogeneous loan, 100 μL of *A. niger* suspension (10^5^ spores/mL) was spread over an empty LBGM Petri dish. Plates were dried thoroughly and uniformly in a biological hood. The overnight culture of *B. subtilis* was either mixed or not with paint. Sterilized Whatman^®^ paper (Sigma Aldrich, St. Louis, MO, USA) was perforated to create symmetrical points for subsequent use. A 1:1 mixture of bacteria and paint (500 μL each) (either anti-fungal paint Supercryl Acrynol—Tambour or a similar paint lacking the fungicide Supercycle) was prepared. Symmetrical points were dipped into the tube containing the color and *B. subtilis* before being placed inside the Petri dishes. *B. subtilis* was inoculated at four symmetrical points, 2.5 cm from the center.

Various Petri dish configurations were arranged, including those with the pathogen alone, *B. subtilis* alone, *A. niger* with *B. subtilis*, *A. niger* with paint, and *A. niger* with paint mixed with *B. subtilis*. Plates were incubated at room temperature. After a 10-day incubation period at 25 ± 1 °C, the diameter of the inhibition zone was measured.

**Antifungal Activities of Microbial Secreted Products.** Antifungal assays were performed in 96-well plates. A 96-well plate was loaded as follows: Sep-Pak concentrated aqueous extracts (50X) obtained as described above were diluted 1:50 in a fresh medium. Then, a volume of 150 µL of the RPMI 1640 medium (Gibco, Waltham, MA, USA) buffered to pH 7.0 with 0.165 M MOPS (Sigma Eldrich) either applied or not with the indicated conditioned media and containing the spore inoculum (described above) was added using Multidrop Combi (Thermo Fischer Scientific, Waltham, MA, USA). Each microplate contained a set of control wells located in the left and right columns. Culture plates were incubated at 37 °C for 24 h. After 18 h of incubation, the data were quantified using optical density (OD) (OD = 600 nm) in a microplate spectrophotometer as in [49] (Infinite^®^ 200 PRO Microplate Reader, Tecan LTD, Männedorf, Switzerland). Inhibition assays were conducted in triplicate and over two independent repetitions. When indicated, the anti-fungal activity of the *B. subtilis* secretions was expressed as the fungal growth inhibitions in percentage (%), after deduction of the relevant negative control (empty medium).

**Image processing.** To quantify the efficiency of *A. niger* inhibition, we developed a new algorithm that measures the ratio of *A. niger* spread for various distances from the spot containing the paint with its anti-mold ingredients. The detailed algorithm can be found in Figure S2. The algorithmic steps are as follows: 1. Identify the relevant area in the image and crop accordingly; 2. Apply a set of thresholds to mark pixels with *A. niger*; 3. Identify the spots with paint and mark their centers; 4. Calculate the ratio of pixels with mold versus the total number of pixels for a few different distances from each disk.

**Growth and Sporulation assays for *B. subtilis*.** To analyze the cell-numbers of *B. subtilis* in paint, the paint–bacteria solution was prepared as described, and incubated as indicated. After robust vortexing, cells were treated as described in the figure legends, serially diluted as carried out by us previously [47] and plated to manually count the emerging colonies.

**Statistical Analysis.** The statistical analysis was performed using GraphPad software. Anova multiple comparisons test was performed for the number of repeats and treatments described under each figure legend.

## 3. Results

### 3.1. Antifungal Effects Are Achieved Using Multiple Biosynthetic Clusters

**Figure 1:**
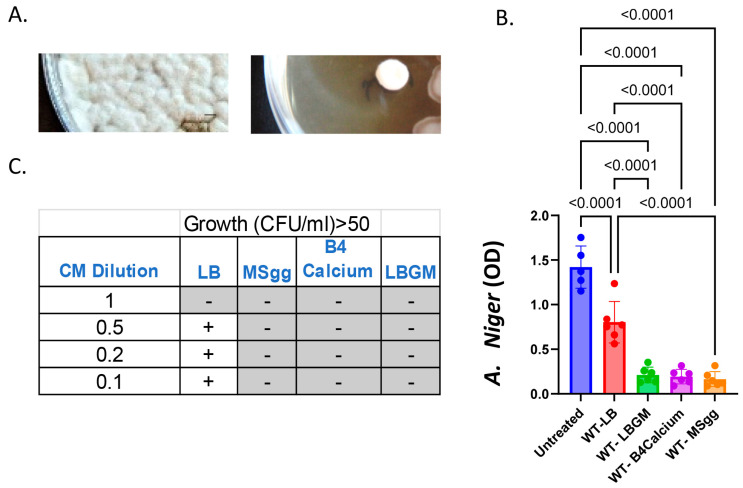
**Anti-mold activities of *B. subtilis*.** (**A**) The growth of *A. niger* colony (white) is limited when grown either in isolation (left) or next to *B. subtilis* (brown) (right) on LBGM medium. Scale bar: 2 mm. (**B**) The conditioned media were collected from *B. subtilis* grown overnight in the indicated media. The collected growth media were concentrated (x50) on C-18 sep-pack and extracted with methanol. The extract was diluted to 1:50 in RPMI medium after 18 h, as carried out previously [50]. For more information, see Materials and Methods. The graphs represent mean and standard deviation of six independent repeats. (**C**) The conditioned media from *B. subtilis* grown overnight in indicated media were collected, and concentrated (x50) on C-18 sep-pack column prior to its extraction with methanol. The *A. niger* cells were diluted in either the conditioned media or with conditioned medium diluted in a RPMI medium to the indicated concentrations (CM dilution). Cultures were plated after 18 h, as carried out previously [50]. For more information, see Materials and Methods. The graphs represent the results obtained with 3 independent repeats carried out in duplicate. Grey [44,47] indicates no colonies were observed. [+] Colonies were observed at 10^−1^ dilution.

*A. niger* is a fungal mold classified within the Nigri section of the Aspergillus genus. The members of this genus are found throughout the environment. They are ubiquitous in fecal matter, soils, water bodies, decomposing matter, and even spore suspensions in the atmosphere. Therefore, we first systematically assessed the effects of the undomesticated *B. subtilis* strain versus this mold model organism on a rich growth medium optimized for the biofilm formation of *B. subtilis* (Figure 1A). This inhibitory effect did not diminish with time.

In suspension, our results indicated that the secreted products of the wild-type *B. subtilis* significantly inhibited the growth of *A. niger* (Figure 1B). This effect was observed similarly on all rich biofilm-inducing media, rich, e.g., LBGM, B4-Ca [46] and a defined medium (MSgg [47]). While an antifungal effect (about 40%) was obtained with the conditioned medium obtained from the LB (Luria Bertani) medium, it was significantly smaller than the effects in LBGM, BR-Ca, and MSgg (Figure 1B). The results were also evident when the colony-forming units of A. niger treated with different dilutions of the conditioned medium of the bacterium from each condition were tested (Figure 1C). Previous findings indicated that the rich medium LB does not allow the transcriptional activation of genes associated with biofilm development [44,51]. Therefore, we tested whether the significant inhibition of *A. niger* can be attributed to one or more previously described anti-fungal biosynthesis clusters, associated with biofilm formation [24].

The pH of the conditioned media was measured previously (refs) and could not account for the toxicity: MSgg is buffered to 7 with phosphate buffer [47], the conditioned medium from B4-Ca is slightly alkaline due to Urease production [46], and the conditioned medium from LBGM is somewhat acidic (6.5 ± 0.3 following the growth period), as *B. subtilis* does not ferment heavily under these conditions [48].

**Figure 2:**
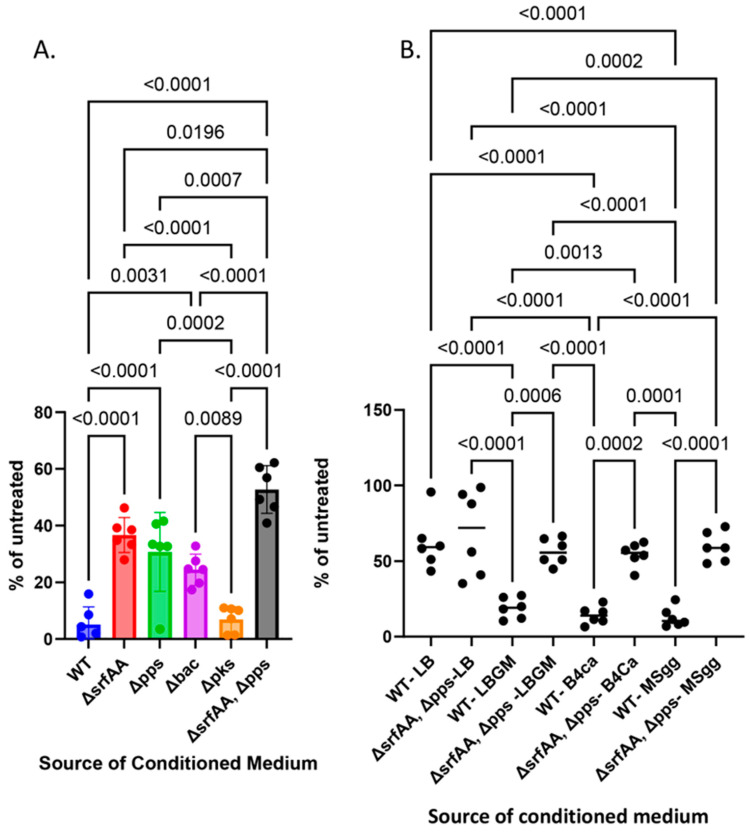
**Anti-mold activities of *B. subtilis* are due to the synergistic activation of anti-fungal biosynthetic clusters.** The conditioned media from *B. subtilis* and its mutant derivatives grown overnight in LBGM (**A**) or indicated growth media (**B**) were collected and tested for the inhibition of *A. niger* as described in Figure 1. The graphs represent the mean and standard deviation of six independent repeats.

While the many clusters of secondary metabolites of *B. subtilis* were previously characterized for antifungal activity, their activities across biofilm growth media and the potential for synergy between the clusters for antifungal activities remained to be determined systematically. From single mutants, we could identify a significant decrease in the inhibitory capacities of mutants of surfactin production (∆*srfAA*), plipastatin (∆*pps*), and Bacilysin (∆*bac*), but not of a mutant defective in bacillaene production (∆*pks*). We then assessed the potential synergy of the two surfactants, surfactin and plipastatin. Indeed, a double mutant for surfactin production (∆*srfAA*), and plipastatin (∆*pps*) lost most, although not all, of its inhibitory properties versus *A. niger* (Figure 2A, Figures S1 and S2). The antifungal effect was significantly diminished compared with each of the single mutants. We found that the mechanisms of fungal inhibition were also comparable between the different growth media, indicating the robustness of their effects (Figure 2B).

### 3.2. Bacillus subtilis Can Robustly Enhance the Anti-Mold Properties of Commercial Paints

**Figure 3:**
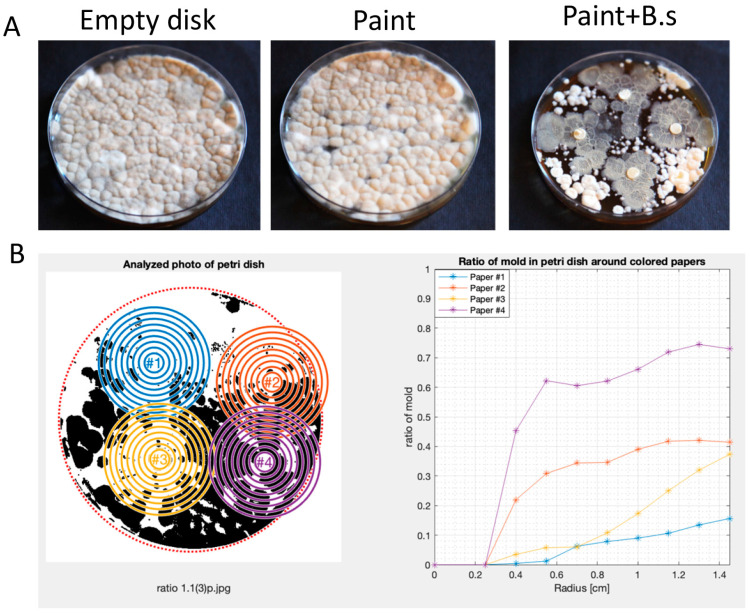
**Anti-mold activities of *B. subtilis* are maintained in paint.** (**A**) Whatman disk was either unsoaked (empty disk) or soaked in commercial paint diluted with water 1:1 (paint) or soaked in commercial paint diluted with *B. subtilis* overnight culture (paint + B.s) as indicated. (**B**) Image analysis of the fungal growth.

Our findings that *B. subtilis* produces multiple anti-mold agents led us to test the introduction of this agent into paint as an effective anti-mold agent. For these purposes, we mixed equal proportions of commercial paint (Tambur LTD) that was not enhanced with chemical antifungal agent. We soaked Whatman Disks in paint and covered them with *A. niger* spores’ solution. As shown, in the absence of *B. subtilis*, *A. niger* grew around and on top of the paint undisturbed (Figure 3A) but failed to do so when the bacterial culture was mixed 1:1 with paint.

To better quantify the effect, we developed a MATLAB script designed for the automated analysis of mold growth in Petri dishes based on high-resolution photos (Figure 3B). The code employs image processing techniques to recognize Petri dishes, quantify mold growth, and visualize the results (Figure S3).

**Figure 4:**
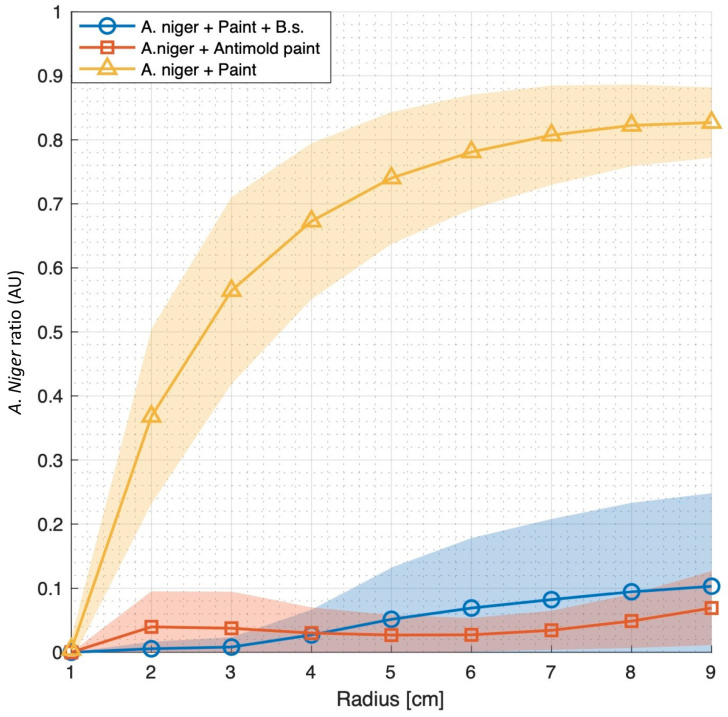
**Quantification of anti-fungal activities of *B. subtilis*-based paint.** Commercial anti-mold or commercial paint diluted with *B. subtilis* was quantified as described in the supporting information (Figure S3). Experiments were carried out with six independent repeats. Results are of independent technical repeats for Figure 3.

The process is shown in Figure 3B and detailed in supporting information. In short, the algorithm was based on high-resolution images of mold *B. subtilis* interactions, calculating mold growth in treated and untreated samples as a function of the distance of the mold from the paint measured in cm. [For details, please refer to Materials and Methods] Our results indicated that *B. subtilis* was an effective agent in reducing *A. niger* spread through multiple plates (Figure 3A,B). *B. subtilis* application was even efficient compared with a commercial mold containing the broad-spectrum anti-fungal biocide (Figure 4). Results were reproduced in independent experiments and with multiple technical repeats, suggesting high compatibility of this bacterium as an anti-mold paint additive in industrial settings.

### 3.3. Bacillus subtilis Can Survive as a Viable Agent and Sporulate in Paint Preparative

**Figure 5:**
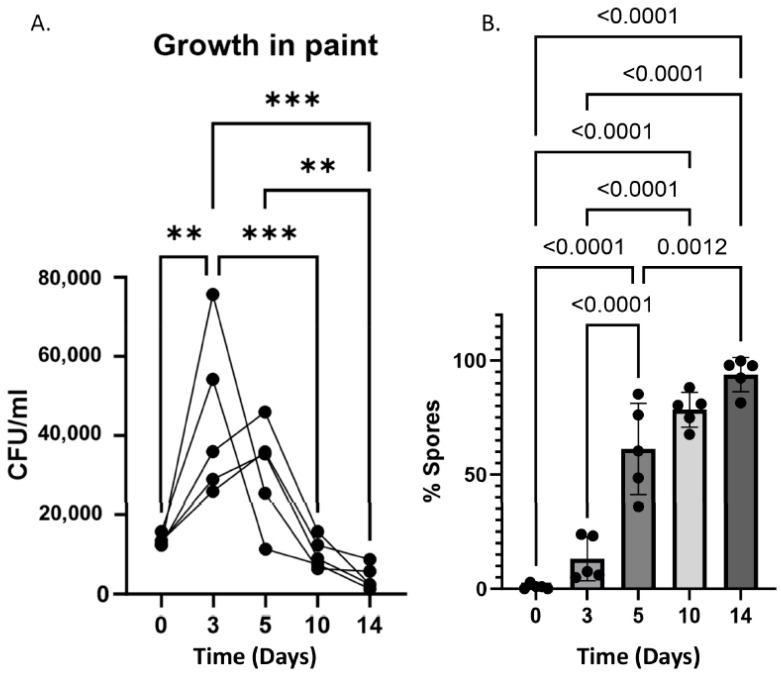
**Viability and Sporulation of *B. subtilis* in paint.** The paint–bacteria solution was prepared at indicated times, as described in Figure 4. After intense vortexing, 1 mL of the solution was taken. A total of 500 µL was mildly sonicated and evaluated for overall CFU numbers/mL in the solution using serial dilutions and plating (**A**). The remaining solution was heat-activated (80 °C, 30 min) to eliminate non-sporulating cells before plating versus the untreated half. The percentage of spore cells was calculated (**B**) by calculating the ratio between spores and untreated cell counts. The graphs represent the mean and standard deviation of five independent repeats. ** represents <0.01, *** <0.001.

Most paints consist of the same essential components: pigments, binders, and additives, in addition to liquid solutions. As we mixed bacterial culture with the paint in a 1:1 ratio, nutrients were available to support bacterial growth further. Consistently, the modest growth of the bacteria was evident for the first three days (Figure 5A). A decline in cell counts was observed after 48 h, indicating that growth within the chemical compounds was suboptimal. As the antifungal activity was mediated by secreted compounds (Figure 2), it is highly feasible that the anti-mold properties of paint were not affected by this decline, as the bacterium produced multiple antimicrobial substances during the initial growth and, therefore, within the paint.

*B. subtil* is undergoes sporulation, a process where the vegetative cells, which are the dividing cells, undergo an asymmetrical cell division to generate a highly resistant cell-type endospore. When conditions become favorable, these spores can re-germinate, allowing the reconstruction of the bacterial biomass [52]. Under our conditions, the efficiency of the process was suboptimal (as the overall cell numbers reflecting vegetative cells and spores declined over time). Based on comparing the results of the colony-forming units’ assay before and after the heat exposure, the total cell numbers after day 5 represented primarily spores (Figure 5B).

## 4. Discussion

The progress in studying microbial genetics opens emerging new fronts for generating live materials with new desired properties. As bacterial secondary metabolites with antibacterial and anti-fungal properties are identified, the utilization of their producers to enhance the antibacterial activities of surfaces becomes more reasonable in different biotechnologies. For example, the *Bacillus* antimicrobial lipopeptide potent plipastatin was suggested to replace conventional anti-fungal substances in agriculture. Recently, *B. subtilis* 3NA (a non-sporulation strain) was engineered, among other methods, using a markerless substitution of the comparably weak native plipastatin promoter (P_pps_). This weak promoter was replaced with a strong constitutive promoter P_veg_ under the control of the housekeeping sigma factor (SigA). However, additional changes were introduced to this strain to confirm the mono-production of plipastatin, potentially reducing potential synergistic drug interactions between plipastatin and other anti-fungal agents produced in this bacterium [53].

The construction industry exemplifies a traditional sector where the potential utilization of bacterial-based technologies is underdeveloped. In this industry, one major obstacle in sustainable construction is the generation of surfaces that can effectively repel and inhibit mold development. Today, one issue is that the chemical substances utilized to prevent mold development are broad-spectrum chemicals that present an environmental hazard rather than an environmentally friendly solution.

*B. subtilis* is an optimal candidate for anti-mold paint, as it generates mineralized layer, with antibacterial and antifungal properties [54]. A significant part of the genome of this bacterium (4%) is dedicated to making secondary metabolites [55], including broad-spectrum antibacterial and antifungal agents [8]. In our work here, we first wanted to understand whether different antimicrobial substances produced by this bacterium can collectively contribute to efficiently inhibiting mold development, as judged by the model mold-forming fungi, *A. niger*. *B. subtilis* produces three potent non-ribosomal peptides, surfactin, bacilysin, and plipastatin (fungycin), analyzed here as anti-mold agents, with little or no contribution of the polyketide bacillaene to the anti-fungal activities.

While we previously demonstrated the hierarchy of these clusters, of the antagonistic activities of these clusters towards *firmicutes*, it remains unknown whether these antibiotics serve as anti-mold agents and whether their production contributes additively to this activity. Here, we found that the output of NRPs but not PKS antibiotics was explicitly utilized to antagonize *A. niger* effectively. Our results demonstrated that NRPs antibiotic surfactin and plipstatin (fungi) act synergistically, but bacilysin also contributes to the antifungal activities in multiple growth media (Figure 2B).

For *B. subtilis* and related species, both antagonistic and non-antagonistic interactions were reported with *Aspergillus species* [56,57]. These results could be due to the context-dependent activation of relevant biosynthetic clusters, as we found that *B. subtilis* can effectively eliminate mold-associated *A. niger* once grown in a biofilm medium but not LB. Consistently, we and others demonstrated the expression of multiple NRP antibiotics under biofilm growth conditions [24,30,58] and their activation by the phosphorylated biofilm inducers DegU-P and Spo0A-P [59].

We also found robustness in the effect of the clusters across three seemingly unrelated growth media: MSgg, a defined medium with glutamate as a nitrogen source and glycerol as a carbon source [44,47,60]; LBGM, a rich medium with tryptone extract as the primary carbon source [61]; and the supplementation of yeast extract and glycerol as a primary carbon source, and B4-Ca where yeast extract serves as a limited carbon and nitrogen source [46]. In these media, the core metabolism and pH are different. However, biofilm genes are robustly activated. The robustness of the anti-mold effects of the identified clusters and the reproduction of synergism between the *pps* and *srfAA* clusters (Figure 2B) indicate the potential adaptation of *B. subtilis* biofilms to fungal treatments in their natural environment or as a pre-requirement for the accumulation of communication factors or local bacterial density to activate these biosynthetic clusters properly.

Our findings that *B. subtilis* can efficiently replace chemical supplements to generate anti-mold paint (Figure 4) highlight the advantage of a living agent as an anti-mold substance, producing multiple (here, at least three) active agents simultaneously and thereby reducing the change for the emergence of antibiotic resistance (Figure 1, Figure 2, Figures S1 and S2). This may especially be of importance to the competitiveness of this bacterium in the plant rhizosphere and its potential contribution as an antifungal biopesticide in agriculture [8,62]. We also demonstrate that the optimization of growth conditions to increase the production of anti-fungal molecules is essential for optimizing effectivity.

The anti-mold activity of *B. subtilis* in paint is not trivial. First, it indicates that the chemical and biological properties of the products of *srf* and *pps* operons are maintained in the complex commercial solutions. Second, it allows a living agent to be integrated into the paint. As sporulation commences (Figure 5) within the paint, re-activating anti-mold activities by germinating the spores is feasible. These unique aspects are significant as current anti-mold paints are associated with environmental toxicity. They are based on the incorporation of living agents rather than non-renewable chemicals whose activities decay over time [63]. The prolonged survival of *B. subtilis* spores, estimated to be at least hundreds of years [64], not only provides advantages in terms of mold prevention but also offers a hopeful prospect for sustainable solutions. These solutions may not be limited to *B. subtilis* and can be easily extended to most sporulating beneficial bacteria of the Bacillus genus and others capable of producing multiple antifungal substances simultaneously. Moreover, the efficient utilization of *B. subtilis* cultures to enhance the anti-mold properties of paints opens an opportunity window for synthetic biology: augmenting the production of these clusters by the removal of their known repressors (for a detailed summary, see [8,53,65]), or an approach based on bio-convergence where the expression and delivery of these antibiotics, as well as the survival of the bacteria, will be optimized. Notably, even in the absence of optimization, we could demonstrate robust effects of *B. subtilis* as an anti-mold agent (Figure 4), capable of sporulating in high concentrations of commercial paint (Figure 5). These results strongly support that an approach of gradually replacing the anti-mold components of paint with probiotic bacteria is an applicable and appealing approach. Moreover, in 2015, the United Nations issued an urgent call exemplified by 17 sustainable development goals (SDGs) to improve health and education while tackling climate change [66]. Goal 11 calls explicitly to “make cities and human settlements inclusive, safe, resilient and sustainable”. Within this framework, the improvement of the built environment, as well as the generation of sustainable live bio-paints, becomes a cardinal milestone.

## Data Availability

All relevant data is included within this manuscript.

## Supplementary Materials

The following supporting information can be downloaded at: https://www.mdpi.com/article/10.3390/microorganisms12061226/s1.
