# Supplementary material for: The Utilization of Bacillus subtilis to Design Environmentally Friendly Living Paints with Anti-Mold Properties
Source: Microorganisms. 2024 Jun 18;12(6):1226. doi: 10.3390/microorganisms12061226 (PMC11205451; doi:10.3390/microorganisms12061226)
Supplement: Supplementary file 1 [file microorganisms-12-01226-s001.zip › microorganisms-2994171-supplementary.pdf]

The utilization of *Bacillus subtilis* to design environmentally friendly living paints with anti-mold properties.

Yuval Dorfan[$1], Avichay Nahami[1,2*], Yael Morris[1*], Benny Shohat[1*], Ilana Kolodkin-Gal[$2]

[1] Faculty of Electrical Engineering, Holon Institute of Technology, Holon, Israel

[2] The Scojen Institute for Synthetic Biology, Reichman University

Supporting Figures:

Figure S1-3.

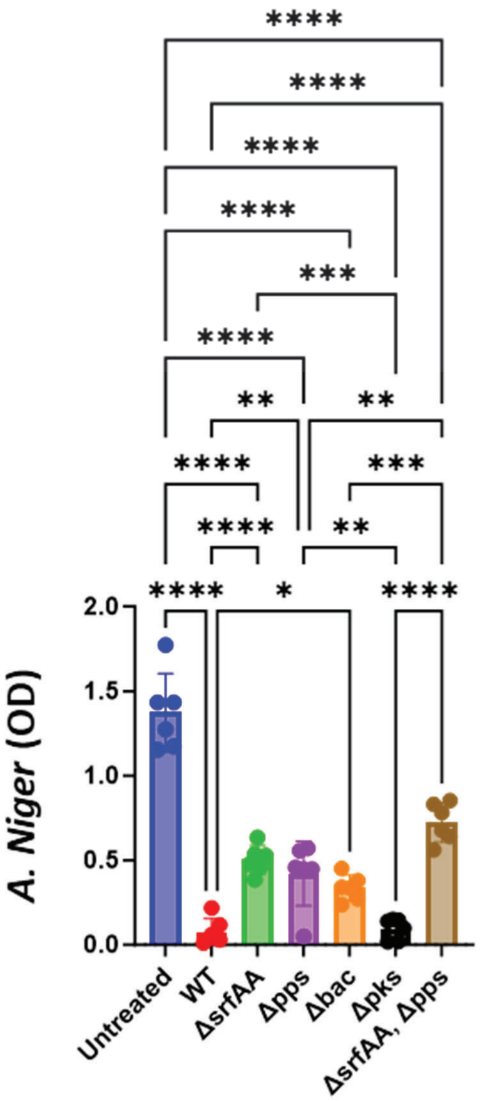

Figure S1. Absolute OD_{600} values after reducing the negative control (empty growth media) for Fig. 2A. Resultes represend the average and standard deviation of the independent biological repeats. * Indicated P-value. * <0.05, ** <0.01, ***<0.001, ****<0.0001

	Growth (CFU/ml)>50				
**CM Dilution**	**WT**	***ΔsrfAA***	***Δpps***	***Δpks***	***Δbac***
1	-	-	-	-	-
0.5	-	Variable	+	-	+
0.2	-	+	+	-	Variable
0.1	+	+	+	+	+

Figure S2. The conditioned media from *B. subtilis* grown overnight in LBGM or of its mutant derivatives grown under the same conditions was collected and concentrated (x50) on the C-18 sep-pack column before its extraction with methanol. The *A. niger* cells were diluted in either the conditioned media or with conditioned medium diluted in an RPMI medium at indicated concentrations (CM dilution) and plated after 18 hours, as done previously [1]. For more information, see materials and methods. The graphs represent the results obtained with three independent repeats done in duplicates. Grey [-] no colonies were observed [+] Colonies were observed at 10^{-1} dilution.

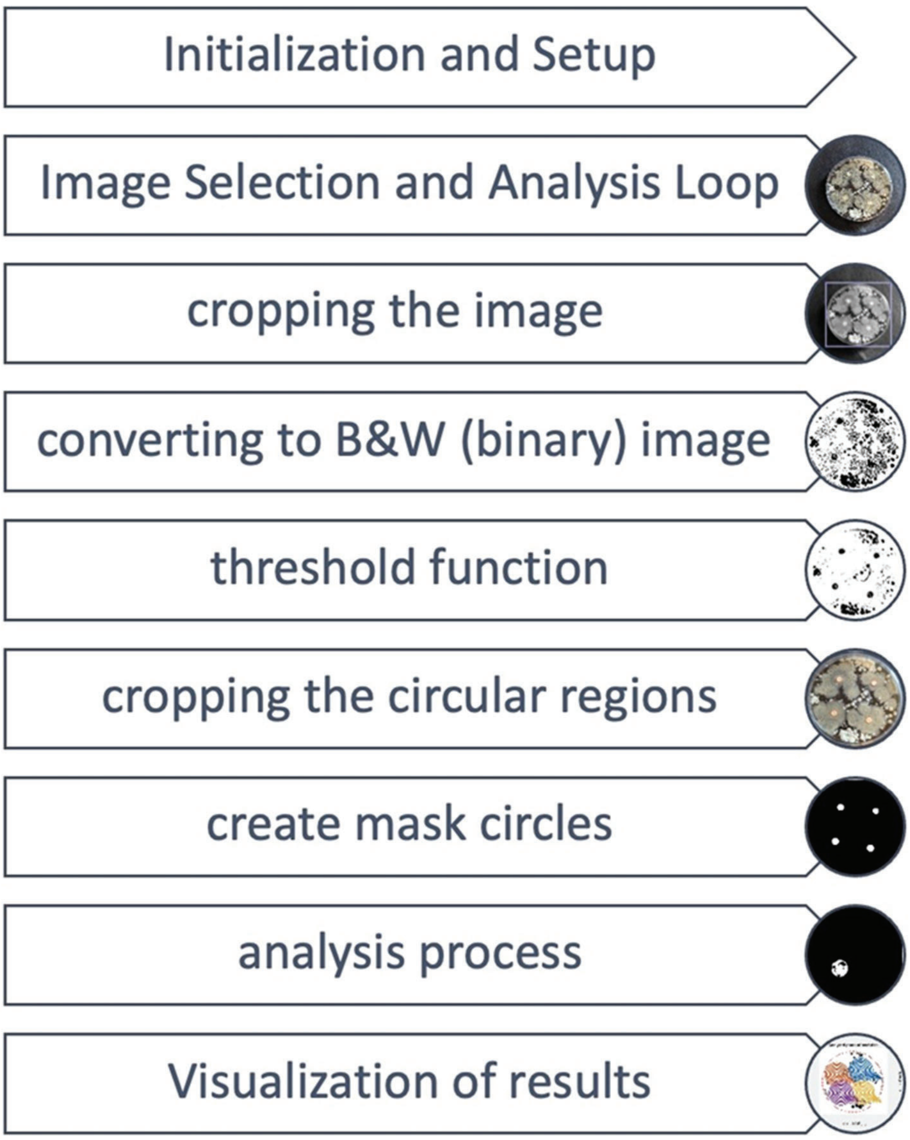

Figure S3. Algorithmic software script structure for image analysis.

Supporting Reference:

1. Miyazawa K, Umeyama T, Hoshino Y, Abe K, Miyazaki Y: **Quantitative Monitoring of Mycelial Growth of Aspergillus fumigatus in Liquid Culture by Optical Density.** *Microbiol Spectr* 2022, **10:**e0006321.
